# A bibliometric analysis of cultural heritage research in the humanities: The Web of Science as a tool of knowledge management

**DOI:** 10.1057/s41599-023-01582-5

**Published:** 2023-03-06

**Authors:** Ionela Vlase, Tuuli Lähdesmäki

**Affiliations:** 1 Lucian Blaga University of Sibiu, Sibiu, Romania; 2 University of Jyväskylä, Jyväskylä, Finland

**Keywords:** Complex networks, Sociology

## Abstract

Substantial research on the topic of cultural heritage has been conducted over the past two decades. At the same time, the overall output volume of journals and citation metrics have become important parameters in assessing and ranking researchers’ performance. Even though the scholarly interest in cultural heritage has recently increased world-wide, a comprehensive analysis of the publication output volume and its correlation to the shift in the cultural heritage regime starting in 2003 is still lacking. The article aims to understand the role of Web of Science (WOS) as a tool of knowledge management in academia by drawing on the scholarly output volume, the patterns displayed by this volume, and the intellectual structure of cultural heritage research based on WOS-indexed journal articles. The data include 1843 journal articles published between 2003 and 2022 and indexed in the WOS Core Collection. The article draws on a bibliometric analysis by using WOS tools and employing VOSviewer software to map and visualize hidden patterns of research collaboration and avenues of knowledge progress. The cultural heritage research indexed in WOS was found to be Eurocentric, corresponding to the increasing funding provided by European national and supranational agencies for research funding. Although the indexed research has grown significantly, the bulk of studies on cultural heritage in WOS is concentrated in a reduced number of European institutions and countries, written by a small number of prolific authors, with relatively poor collaborative ties emerging across time between authors, institutions, and countries. The central themes reflect the development of digital technologies and increased participatory emphasis in cultural heritage care. This article brings new insights into the analysis of the cultural heritage research in correlation with the emergence of international heritage governance with new institutional actors, professional networks, and international agreements, which are all constitutive elements of scientific production. The article seeks to critically assess and discuss the results and the role of WOS as a tool of knowledge management in academia.

## Introduction

Cultural heritage is a multidisciplinary topic that has garnered increasing scholarly interest world-wide during the past few decades (Waterton and Smith, 2009; Harrison, 2013b; Lähdesmäki et al., 2020; SoPHIA, 2020). This interest is reflected in the launch of new research centers, study programs, scholarly associations, conference and seminar series, and research projects that go beyond the traditional view of cultural heritage as material objects requiring conservation and preservation. Publication has a central role in strengthening cultural heritage scholarship: more and more studies are published in peer-reviewed journals on topics ranging from natural to social sciences and from education to the humanities. The increase of publications on cultural heritage across a wider range of publication fora corresponds to the general increase of publication output volume in academia (see for a general tendency, e.g., Kyvik and Aksnes, 2015; Fire and Guestrin, 2019). Cultural heritage scholarship is not only on its way to become international, but also seeks to improve its quality according to productivity metrics and quality assessment methods borrowed from natural sciences.

Even though scholarly interest in cultural heritage has recently strengthened and extended to cover a broad spectrum of topics, the scholarship’s tradition has its roots in the humanities, more particularly in the rise of antiquarianism, the emergence of archeology, and the collecting of antiquities and other rarities in fifteenth- and sixteenth-century Europe. This history is well documented in the literature (see e.g., MacGregor, 2007; Bahn, 2014; Miller, 2017). Since the early nineteenth century, research on cultural heritage has served various nationalist agendas: histories of cultural heritage and practices of exhibiting and conserving it have played (and often still play) an important role in the construction of nation-states and national identities (see e.g., Gillis, 1994; Aronsson and Elgenius, 2015; Huber, 2021). In the twentieth century, the destruction of material cultural heritage during the World Wars and, after the wars, the emergence of international heritage governance with new institutional actors, professional networks, and international agreements such as UNESCO and its heritage conventions stimulated professional interest in heritage conservation and legislation. The institutional and legislative development of the field has been broadly explored in the literature (see e.g., Bendix et al., 2013; Swenson, 2016; Meskell, 2018). At the end of the twentieth century, cultural heritage scholarship was broadened by new critical approaches. For instance, the established uses of cultural heritage were criticized by several researchers scrutinizing how nationalist sentiments and collective identities have been created through the invention of traditions (Hobsbawm and Ranger, 1983), the fabrication of heritage myths (Lowenthal, 1985), or by cultivating nostalgia for the past (Hewison, 1987). In the following decades, cultural heritage research was enriched by advancements in memory studies (e.g., Macdonald, 2013; van Huis et al., 2019) and the investigation of previously un-told or marginalized histories and heritage narratives of minorities and indigenous people (e.g., Seglow, 2018). Such interests reflect the critical turn in heritage studies starting with the 2000s, as we will show in the present article. During the past two decades, the scope of cultural heritage research has continued to expand when scholars have approached it as a discursive and performative practice (e.g., Smith, 2006; Waterton and Smith, 2009; Lähdesmäki et al., 2019), an affective and embodied experience (e.g., Waterton and Watson, 2015), and/or emphasized its social, societal, political, ideological, economic, and touristic meanings (e.g.; Ashworth et al., 2007; Chirikure et al., 2010; Zhu, 2021; Lähdesmäki et al., 2020). Moreover, recent research has explored cultural heritage as a source of individual and social well-being (e.g., Wallace and Beel, 2021), an asset for equal, inclusive, and fair societies (e.g., Silverman and Ruggles, 2007; Logan, 2012), and a key for sustainable futures (e.g., Harrison et al., 2020).

The development of cultural heritage scholarship is reflected not only in the extended scope of research and in the sheer quantity of publications, but also in the publication’s scholarly quality and recognition in international publication indexes. Among research databases, for instance, ProQuest Central yields 30,809 results in a field search for peer-reviewed publications with the search term ‘cultural heritage’. Only a small portion of these publications is, however, included in the most established and highly ranked scholarly journal database, such as the Web of Science (WOS).

WOS is one of the core global providers of knowledge, encompassing more than 50,000,000 articles covering 250 scientific categories and about 150 research areas; the articles’ performance is assessed through different indicators, quantifying their impact in terms of citations (Cancino et al., 2017). Since the 2000s, publication output volume and citation metrics have become important parameters in assessing and ranking academic researchers’ performance (Fischer et al., 2012; Fire and Guestrin, 2019; Wahid et al., 2022), even though scholars are aware of the shortcomings of these metrics (Wilsdon, 2016).

Even though cultural heritage scholarship includes a broad body of literature, as well as literary reviews on more specific topics, such as the social and economic value of heritage (Dümcke and Gnedovsky, 2013), the societal impact of cultural heritage (SoPHIA, 2020), climate change and cultural heritage (Orr et al., 2021), contested heritage (Liu et al., 2021), or heritage diplomacy (Lähdesmäki and Čeginskas, 2022)—to mention just a few—the scholarship still lacks a comprehensive analysis exploring publication volume and its patterning in relation to structural forces such as the change in cultural heritage regimes and the emergence of new powerful institutional actors that shape this scientific production. The core objective of our article is to uncover such patterns and understand the intellectual structure of cultural heritage research that is regarded as high-quality due to its presence in one of the most respectable journal databases. Our article contributes to cultural heritage research by providing knowledge on the recent evolution of publication volume and the particularities of this output while paying attention to the cultural heritage regime structuring the intellectual field in heritage studies (Bourdieu, 1983). Moreover, our article contributes to the research of knowledge production by underlining the position of journal databases and the information they collect and provide as a means of producing and structuring knowledge. We understand WOS as a knowledge management tool in academia. As one of the core global providers of bibliometric data, WOS identifies, organizes, and disseminates information on scholarly production and therefore exerts a significant impact on the reputation of different research fields.

In order to reach our objectives, we conducted a bibliometric analysis using WOS ‘analyze results’ and ‘citation reports’ tools to generate descriptive statistics on the growth and impact of 1843 journal articles indexed in WOS. To explore the interdependency of authorship and key topics in the dataset, we then made use of the VOSviewer software to visualize networks of co-authorship and co-occurrences of clustered keywords showing different patterns of research collaborations between authors, institutions, and countries, as well as prominent inter-related lines of inquiry related to cultural heritage. By employing these tools, we seek to illustrate the WOS-indexed evolution of cultural heritage research conducted in the multidisciplinary humanities over the past 20 years.

A wide range of scholars have utilized WOS as a data source for bibliometric studies (Donthu et al., 2021; Crețu and Morândău, 2022; Wahid et al., 2022). These studies indicate how bibliometric analysis can bring out recent thematic tendencies and explain changes in publication output volume to help researchers make informed decisions about their future work (Cancino et al., 2017). However, scholars have noted the limitations of bibliometric analysis and of WOS as a source of data (Holden et al., 2005; Cascajares et al., 2021). One of these shortcomings is that disciplinary differences in the indexation process can have a great influence on citation. Moreover, bibliometric analysis draws on the assumption that citation reflects the quality of the cited source and that all citations are equally important (Poole, 2015), which is not necessarily the case. The humanities were among the last to adopt the bibliometric performance assessment, leading to bibliometric studies in different fields. We identified four studies that have utilized the method in order to explore literature on certain sub-fields or topics in cultural heritage research. Kumar et al. (2020), Bhowmik (2021), and Zhang et al. (2022) have conducted bibliometric analyses to show the development of the main topics in heritage tourism research, as well as its most prominent authors, research institutions, and their host countries. In their article, Zhu et al. (2022) conducted bibliometric mapping and visualization of literature on historical wall paintings, revealing its main thematic focuses and the correlation between the most productive authors and key research institutions. Chen et al. (2020) used the WOS database and CiteSpace bibliometric analysis software in their study in order to explore articles on intangible cultural heritage.

Our article builds on the previous bibliometric research seeking to map, visualize, explain, and understand the publication output volume, the patterns distinguished in the output, and the interdependencies of cultural heritage scholarship. We also draw on previous criticism directed against the method and seek to critically assess the WOS as a tool of knowledge management. The article is structured into five sections. After the introduction, we explain how we built our dataset of bibliometric information for 1843 articles on cultural heritage and describe the growth of this scholarly output over time, across countries, and in the most populated research areas and most used languages. Next, we map the patterns of cultural heritage publications in the humanities to reveal emerging collaborative networks between authors, research institutions, and countries, as well as the most prevalent thematic clusters of cultural heritage research and the recent knowledge-oriented approaches. Subsequently, we discuss the results in the context of the development of cultural heritage research during the past two decades, particularly against the backdrop of its critical turn and the generative matrix of the shifting cultural heritage regime. Finally, we summarize how WOS manages the knowledge on cultural heritage research, discuss the limitations of our study, and suggest future research avenues for scholars working on cultural heritage.

## Data and methodology

To achieve our aim, we selected the most appropriate methods and techniques of bibliometric analysis described by Donthu et al. (2021). Bibliometric analysis is used to detect trends in research evolution within a specific field, to point towards emerging topics shaping the intellectual advancements in that field, as well as to reveal patterns of collaboration among prolific researchers, their countries, and the institutions they are affiliated with. The recent developments in bibliometric analysis allow for the use of various techniques enabling scientists to make sense of large unstructured data that show the growth and impact of relevant publications selected in accordance with the objectives of each bibliometric study, which usually revolve around identifying knowledge gaps within a research topic, informing researchers about the state-of-the-art, and eliciting new research questions. We use common variants of bibliometric analysis such as *performance analysis* and *science mapping*. The former is used to profile relevant research constituents such as authors, institutions, journals, and countries. The latter enables us to understand the relationship between these research constituents by drawing on visual tools such as those provided by VOSviewer software. While performance analysis uses quantitative indicators (e.g., number of total publications, by year, publication, or country, citations per research item), science mapping focuses on patterns of collaboration between selected units (e.g., authors, institutions, countries) to document the social interactions shaping the intellectual structure of research on a topic within a timeframe. In the VOSviewer software, we conducted a co-authorship analysis at the levels of authors, institutions, and countries. Subsequently, we have examined the emerging themes connected to cultural heritage by using co-occurrence analysis based on the assumption that frequently co-occurring keywords bear a strong thematic relationship and therefore, the resulting clusters of these keywords in articles indicate the emergence of subtopics that share an inner consistency as ‘communities of topics,’ characterized by a ‘latent relationship between those topics’ (Emich et al., 2020, p. 662).

Based on these methodological considerations and seeking to understand the evolutionary trends of cultural heritage research across humanities over the last two decades, we collected the data from Web of Science (WOS) by introducing ‘cultural heritage’ in the topic (TS) search field of WOS while maintaining the quotation marks, so that the bibliographic results include this search string as it is instead of separate occurrences of the words ‘cultural’ and ‘heritage,’ that would have yielded results that do not fit the topic of cultural heritage. This selection procedure is informed by recent developments in search strategies using search strings (Ng et al., 2022) and by similar bibliometric research on intangible cultural heritage (Chen et al., 2020). Following such prior studies based on WOS, we have privileged the Topic (TS) search field over the Title (TI) field, which would have limited the results to documents referring to ‘cultural heritage’ in their titles while omitting a large number of relevant studies engaging with cultural heritage that do not mention these search strings in the title but do so within their abstracts or keywords. As our article aimed at mapping the cultural heritage research in the humanities, regardless of its tangible or intangible form, our search strategy ensured that search results cover indexed documents that contain the search string ‘cultural heritage’ within the title, abstract, or keywords. Out of the 27,205 results of the initial query produced on August 5, 2022, when the search was run on the full period covering WOS recorded documents, we have excluded those that were classified by WOS in categories other than humanities by refining the results by using the WOS category filter of ‘Humanities Multidisciplinary’. This filter yielded 2410 documents, almost 9% of those identified by the initial query (see Table 1). Subsequently, we used the WOS document type filter to remove the documents classified other than ‘article’ from the dataset, which reduced our sample to 1,845 entries. Beyond—but increasingly within—the humanities, articles are commonly considered more important scientific contributions than conference proceedings, book reviews, or editorials, and are therefore worth taking into consideration in bibliometric studies (Su et al., 2019).

**Table 1:** The largest Web of Science Categories by number of papers.

Web of Science Categories	Record count	% of 27,205
Humanities Multidisciplinary	2410	8.86
Archeology	2169	7.97
Environmental Sciences	1663	6.11
Architecture	1652	6.07
Materials Science Multidisciplinary	1586	5.83
Computer Science Information Systems	1475	5.42
Geosciences Multidisciplinary	1475	5.42
Art	1440	5.29
Imaging Science Photographic Technology	1354	4.98
Environmental Studies	1336	4.91

Source: Authors based on WOS query results TS = ‘cultural heritage’ excluding keywords plus as of August 5, 2022. *N* = 27,205.

The present article is focused on the development of cultural heritage research over the past two decades, that were marked by the shift in the cultural heritage regime (Cokisler, 2018; Hølleland and Niklasson, 2020). This shift draws on the development in international and national heritage governance and management. During the past 20 years, the core international conventions established by the United Nations Educational, Scientific and Cultural Organization (UNESCO) and charters by International Council on Monuments and Sites (ICOMOS) have provided new guidelines and management principles, for instance, for safeguarding the intangible cultural heritage (by UNESCO in 2003), the preservation of digital heritage (by UNESCO in 2003), interpreting and presenting cultural heritage (by ICOMOS in 2008), the conservation of architectural heritage (by ICOMOS in 2003), industrial heritage (by ICOMOS in 2011), historical urban landscapes (by UNESCO in 2011), and the management of rural landscapes as heritage (by ICOMOS in 2017). Moreover, several international conventions and guidelines, such as the Council of Europe’s so-called Faro Convention (established in 2005), have emphasized the need to better acknowledge the economic and social value of cultural heritage and the significance of promoting citizens’ right to access and participate in heritage. Against this backdrop, national and international bodies have designed governance strategies geared towards the preservation of cultural heritage and the increase of funding dedicated to its research, as reflected in the prominent role occupied by topics related to cultural heritage in the European Union’s recent research and innovation program HORIZON EUROPE for 2023–2024 (EC European Commission, 2022). Acknowledging that the production of scholarly literature on cultural heritage is shaped by powerful structural forces in which researchers are assimilated (Bourdieu, 1983), we only consider articles published after 2003, i.e., that are linked to the turn in the heritage regime, and therefore exclude earlier documents. The final dataset encompasses 1843 articles. Regarding the language, no restriction was applied during the search phases, but the 1843 articles were commonly published in English (71%), followed by Spanish (6.7%) and Russian (6.3%) (see Table 2). For the articles published in other languages, WOS policy requires publishers to also provide titles, keywords, and abstracts in English.

**Table 2:** Most common languages of the sampled documents on cultural heritage.

Languages	Record count	% of 1843
English	1312	71.19
Spanish	123	6.67
Russian	116	6.29
Italian	97	5.26
French	67	3.64
Portuguese	33	1.79
Turkish	23	1.25
Slovak	18	0.98
Slovenian	16	0.87
German	10	0.54

Source: Authors based on based on WOS query results TS = ‘cultural heritage’ classified in the WOS category of Humanities Multidisciplinary. *N* = 1843.

The final search result encompassing 1843 articles was saved as a marked list in WOS. The data was exported as a tab-delimited file using the full record and cited references option to enable subsequent science mapping using the VOSviewer software (version 1.6.16). VOSviewer was used to trace several item clusters (authors, institutions, countries, keywords, or other information from the dataset) based on their high similarity and their dissimilarity with items from other clusters. In the resulting figures, the size of the dots shows the prevalence of an item (e.g., prolific authors, journals, institutions, countries, and frequently co-occurring keywords), while clusters of dots are visually represented through distinct colors. We look at two variables: the co-authorship network at the level of researchers, organizations, and countries, as well as the co-occurrence of author keywords. Therefore, the dots represent authors, institutions, countries, and keywords.

The most populated research areas within Humanities (when looking at the number of publications on cultural heritage in multidisciplinary collaboration with a humanities discipline) are ‘Social Sciences Other Topics’ and ‘Science Technology Other Topics’, while ‘Linguistics’ and ‘Area Studies’ are less popular. The first ten research areas selected in Table 3 account for half of the sampled articles in our dataset.

**Table 3:** Top 10 research areas covered by Multidisciplinary Humanities articles on cultural heritage.

Research areas	Record count	% of 1843
Social Sciences Other Topics	237	12.86
Science Technology Other Topics	168	9.12
Computer Science	148	8.03
Chemistry	107	5.81
Materials Science	107	5.81
Spectroscopy	107	5.81
Linguistics	26	1.41
Information Science and Library Science	12	0.65
Area Studies	8	0.43

Source: Authors based on based on WOS query results TS = ‘cultural heritage’ classified in the WOS category of Humanities Multidisciplinary. *N* = 1843.

## Findings

### The production of scholarly articles on cultural heritage over time

The steady growth of research on cultural heritage over the last 20 years increases exponentially starting from 2017. 1509 articles were published during the past six years (about 82% of the documents included in the final dataset). This growth can be explained as the result of large funding provided by supranational bodies such as the European Commission and other national funders such as UK Research and Innovation (UKRI) and the National Research Council of Italy (CNR), as detailed in the ‘Discussion’ section.

**Fig. 1:**
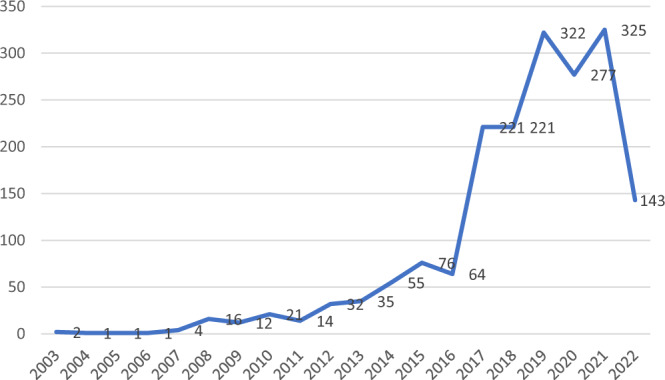
Evolution of number of articles published between 2003 and 2022. Source: Authors based on WOS list of indexed articles 2003–2022 on the topic (TS) of ‘cultural heritage’ published in Humanities Multidisciplinary. *N* = 1843. Articles indexed in by 5th August 2022.

The examination of data by publication year shows two peaks in 2019 and 2021, each with over 320 articles per year (Fig. 1). In order to shed better light on the publications’ performance, we subsequently examine the countries and institutions with a more significant contribution to this growing trend in the scholarly research on cultural heritage. We expect an uneven contribution made by researchers from different countries and organizations, since the tradition of this research in some regions is bound to a rich cultural heritage, including a concentration of historical monuments and archeological sites listed as World Heritage by UNESCO, complemented by expertize in conserving such heritage. Moreover, a few influential scholars have been most productive, and new regional funding opportunities have become available for heritage research.

### Productive countries and institutions

In order to describe the performance of different research constituents on cultural heritage over the past 20 years, we scrutinized the most productive countries and institutions. Table 4 shows the top 10 countries that together account for 69% of the articles in our dataset. Italy, England, and Spain are the most prominent, since one third of the articles are written by authors affiliated with institutions based in these three countries. This result is in line with the expectations drawn from the literature documenting the scientific production in relation to cultural heritage governance prevalently oriented towards safeguarding heritage and sustainable tourism, especially in countries such as Spain and Italy, marked as they are by growing concerns regarding touristification and its consequences; in England, however, the prominent debates revolve around the enhancement of cultural experiences through digitization (Echavarria and Samaroudi et al., 2020).

**Table 4:** Top 10 countries publishing on cultural heritage topic.

Countries	Record count	% of 1843
ITALY	291	15.79
ENGLAND	171	9.28
SPAIN	163	8.84
USA	147	7.98
RUSSIA	126	6.84
FRANCE	104	5.64
AUSTRALIA	81	4.39
GREECE	65	3.53
GERMANY	64	3.47
PEOPLES R CHINA	52	2.82

Source: Authors based on WOS queries using ‘analyze results’ tool on the saved list of *N* = 1843 articles on cultural heritage indexed in the WOS category Humanities Multidisciplinary.

The remarkable contribution made by the University of London and one of its major member institutions, the University College of London (together responsible for 86 articles) is seen in the table listing the most productive institutions (Table 5). These institutions have published at least 15 articles within the past 20 years, which together make up a bit more than a fifth of all sampled articles in our dataset.

**Table 5:** Most productive institutions of affiliation by number of articles.

Affiliations	Record count	% of 1843
UNIVERSITY OF LONDON	49	2.66
UDICE FRENCH RESEARCH UNIVERSITIES	43	2.33
THE NATIONAL RESEARCH COUNCIL CNR	39	2.12
UNIVERSITY COLLEGE LONDON	37	2.01
RUSSIAN ACADEMY OF SCIENCES	23	1.25
THE FRENCH NATIONAL CENTRE FOR SCEINTIFIC RESEARCH CNRS	20	1.08
NATIONAL RESEARCH TOMSK STATE UNIVERSITY	20	1.08
UNIVERSITY OF BOLOGNA	19	1.03
UNIVERSITY OF SEVILLE	18	0.98
SAPIENZA UNIVERSITY ROME	17	0.92
UNIVERSITY OF LJUBLJANA	17	0.92
POLYTECHNIC UNIVERSITY OF MILAN	16	0.87
UNIVERSITY OF FLORENCE	16	0.87
UNIVERSITY OF MACERATA	15	0.81

Source: Authors based on WOS queries using ‘analyze results’ tool on the saved list of *N* = 1843 articles on cultural heritage indexed in the WOS category Humanities Multidisciplinary. About 3.8% of the results in WOS do not contain data on affiliation fields.

### Influential journals and articles

Our dataset includes more than 630 publication titles. The leading position is occupied by the *International Journal of Cultural Heritage*, that accounts for 269 of the sampled articles on cultural heritage. The second most productive publication is *Heritage*, followed by *ACM Journal of Computing and Cultural Heritage*, with 200 and 143 articles, respectively. These three publications account for more than a quarter of the total number of articles included in our sample, while half of the articles are issued by the eight most productive journals listed in Table 6.

**Table 6:** Most productive journals publishing articles on cultural heritage.

Publication titles	Record count	% of 1843	JIF 2021	JIF quartile
*International Journal of Heritage Studies*	221	11.99	1.692	3
*Heritage*	168	9.12	ESCI	n a
*Acm Journal on Computing and Cultural Heritage*	135	7.32	2.047	4
*Heritage Science*	107	5.81	2.843	2
*Capitale Culturale Studies on the Value of Cultural Heritage*	78	4.23	ESCI	n a
*International Journal of Intangible Heritage*	74	4.02	AHCI	n a
*Vestnik Tomskogo Gosudarstvennogo Universiteta Kulturologiya i Iskusstvovedenie Tomsk State University Journal of Cultural Studies and Art History*	58	3.15	ESCI	n a
*Historic Environment Policy Practice*	48	2.60	AHCI	n a

WOS dataset, *N* = 1843. Journal Impact Factor and corresponding quartiles are provided for the most recent year (2021) according to the Journal Citation Report (JCR) in WOS.

The 524 articles published in the three most productive journals amassed a total of 2977 citations in WOS, which represent almost half of the total citations recorded for the whole dataset. Therefore, a little more than a quarter of the sampled articles (i.e., 28.4%) accounts for the 49% of citations, suggesting that these journals are the most influential in cultural heritage. It is worth mentioning, however, that the journals which ensured the highest total citation count contain words related to heritage in their title. For instance, the *International Journal of Heritage Studies* with an Impact Factor of 1.692, ranked in Q3 in Social Sciences Interdisciplinary, gathered a total of 2493 citations, while *ACM Journal on Computing and Cultural Heritage* (IF 2.356, Q4 Computer Science, Interdisciplinary applications), and *Heritage Science* (IF 2.843, Q2 Spectroscopy), produced 1,083 and 604 citations, respectively. These journals scored higher average citations per published paper (i.e., 8, 11, and 6, respectively) compared with the average citation of 3.34 of the full article dataset. This finding suggests that authors looking for higher exposure of their research on cultural heritage could seek to publish in journals with the word ‘heritage’ in the title.

The articles in WOS with the highest number of citations are listed in Table 7. The two leaders are articles jointly written by five and four authors, affiliated to different organizations based in different countries. The most cited paper (202 citations) is entitled ‘A Survey of Augmented, Virtual, and Mixed Reality for Cultural Heritage’ (Bekele et al., 2018) and was published by five authors affiliated with institutions from three countries (University of Cape Town in South Africa, Curtin University in Australia, and Marche Polytechnic University in Italy) published in 2018 by the *ACM Journal of Computing and Cultural Heritage*, the journal with fifth highest mean citation (8) per article on cultural heritage. This higher impact needs to be contextualized with respect to the average number of authors per article in this field. About 48% of the articles in our dataset are authored by one researcher, another 21% have two authors, while 31% of the sampled documents have three or more authors.

**Table 7:** The most cited articles on cultural heritage.

Authors	Article title	Source title	Number of citations
Bekele MK et al. (2018)	A Survey of Augmented, Virtual, and Mixed Reality for Cultural Heritage	*ACM Journal of Computing and Cultural Heritage*	202
Chirikure S et al. (2010)	Unfulfilled promises? Heritage management and community participation at some of Africa’s cultural heritage sites	*International Journal of Heritage Studies*	103
Harrison R (2013)	Forgetting to remember, remembering to forget: late modern heritage practices, sustainability and the crisis’ of accumulation of the past	*International Journal of Heritage Studies*	97
Winter T (2014)	Beyond Eurocentrism? Heritage conservation and the politics of difference	*International Journal of Heritage Studies*	66
Holtorf C (2015)	Averting loss aversion in cultural heritage	*International Journal of Heritage Studies*	65
Roberts L & Cohen S (2014)	Unauthorising popular music heritage: outline of a critical framework	*Heritage Science*	65
Logan W (2012)	Cultural diversity, cultural heritage and human rights: towards heritage management as human rights-based cultural practice	*International Journal of Heritage Studies*	62
Leissner J et al. (2015)	Climate for Culture: assessing the impact of climate change on the future indoor climate in historic buildings using simulations	*Heritage Science*	58
Rubino I et al. (2015)	Integrating a Location-Based Mobile Game in the Museum Visit: Evaluating Visitors’ Behavior and Learning	*ACM Journal of Computing and Cultural Heritage*	57
Mydland L & Grahn W (2012)	Identifying heritage values in local communities	*International Journal of Heritage Studies*	57

WOS dataset of articles on TS = cultural heritage published 2003–2022. *N* = 1843.

The author with the highest number of articles on cultural heritage in our dataset is Massimo Montella, University of Macerata (Italy), who authored 12 articles, along with other various types of texts written during the past decades and published online in *Il Capitale Culturale: Studies on the Value of Cultural Heritage* in 2020. He specializes in economics, heritage marketing, the theory of cultural heritage management, and cultural heritage as service (see e.g., Montella, 2020). Melissa Terras from the University of Edinburgh, Scotland, is the second most prolific author, having published seven joint articles in two journals (i.e., *Digital Scholarship in the Humanities* and *Big Data and Society*) between 2017 and 2021, with 53 citations. Her expertize is in digital cultural heritage and her recent work exposes the dissimilarities between some Western European and Russian policies of open access to digitized museum objects (see, e.g., Terras et al., 2018).

### Collaborative ties between prolific authors

**Fig. 2:**
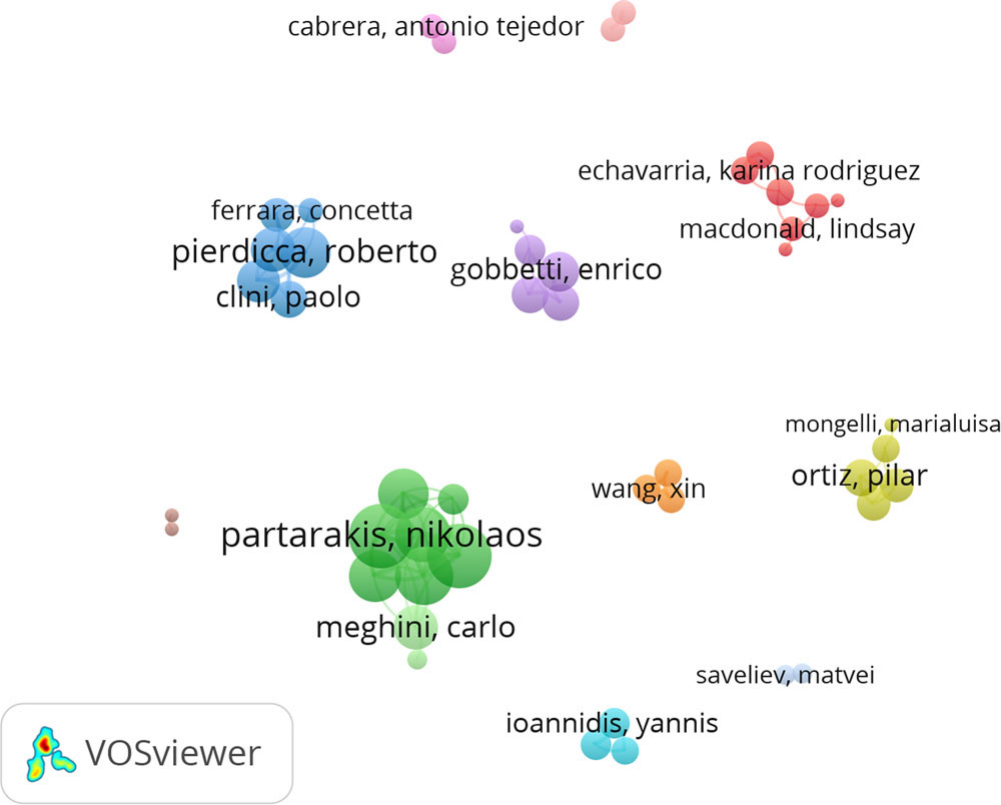
Co-authorship analysis at author level. Note: Minimum number of papers per author *n* = 3, number of authors meeting this threshold *n* = 78. The authors represented in the map (*n* = 46) have a total link strength of at least 1 and are grouped in 12 clusters in different colors.

Despite the small number of prolific authors with three articles or more (78), we further mapped the strength of links between authors based on the direct collaboration through joint publications. After creating a thesaurus file on authors to eliminate duplicates from the dataset by merging different spellings of the same name, we used VOSviewer to perform a co-authorship analysis for the 78 authors who met the criteria of having published at least three articles on cultural heritage. For those authors, a total link strength was calculated using the full counting method of co-authorship ties between two authors (with a total link strength score ranging from a minimum of 1 to maximum 21). No connection was found for 32 prolific authors. For the remaining 46 prolific authors, VOSviewer mapped the emergence of 12 clusters that indicate distinct patterns of collaboration (Fig. 2).

The green cluster groups together seven authors from a more territorially bounded institutional setting: Ilia Adami, Danai Kaplanidi, Effie Karuzaki, Sotiris Manitsaris, Nikolaos Partarakis, Xenophon Zabulis, and Emmanouil Zidianakis. With only two exceptions (i.e., two other Greek speakers, Manitsaris from PSL Research University Paris and Kaplanidi from Piraeus Bank Group Cultural Foundation, Greece), the authors in this cluster are all affiliated with the Foundation for Research and Technology—Hellas, Greece. Partarakis and Zabulis are the most connected researchers of this group. They have both co-authored several papers with other researchers from our dataset (e.g., scholars from research institutions based in Germany, Switzerland, France, and Italy). These collaborations resulted in a total link strength of 21, indicating strong collaborative ties for each of them. Karuzaki is the next most connected researcher from this cluster, with a total link strength of 17, followed by Adami and Kaplanidi, with 15 and 14, respectively. They wrote joint articles on topics including the representation of the traditional craft and its transfer in the museum and the role of recipes in the culinary tradition (e.g., Partarakis et al., 2021). This analysis on collaborations carried out at the author level suggests that co-authors work in small groups, most commonly affiliated with the same institutions.

Another sizeable cluster in red encompasses seven authors from four different institutions, namely the University of Brighton (Karina Rodriguez Echavarria and Myrsini Samaroudi), University College London (UCL, Lindsay MacDonald, Melissa Terras, and Tim Weyrich), Norwegian University of Science & Technology (Pillay Ruven), and Durham University (Claire Warwick), who co-authored papers on the esthetic judgment of Spanish art through eye tracking the visual reactions of people exposed to Francisco de Zurbaran’s paintings in a laboratory setting (e.g., Bailey-Ross et al., 2019). Within this cluster, the highest total link strength of four is recorded for three authors, namely Echavarria, Samaroudi, and Weyrich, the rest of the authors being less connected with other scholars publishing on this topic. In 2020, Echavarria and Samaroudi co-authored ‘Heritage in lockdown: digital provision of memory institutions in the UK and US of America during the COVID-19 pandemic’ published in *Museum Management and Curatorship*, that has received 29 citations in WoS, the most cited paper from this cluster.

The third most populated cluster of co-authors in blue is made up by six researchers, namely Paolo Clini, Emanuele Frontini, Marina Paolanti, Roberto Pierdica, and Ramona Quattrini, all affiliated with Marche Polytechnic University (Italy), and Ferrara Cocetta from University of Macerata (also Italy). Roberto Pierdicca had the highest link strength in this cluster, 13. The role of digital technologies such as apps in the promotion of tourism is one of the contributions of these authors to cultural heritage (e.g., Clini et al., 2019).

The next cluster in yellow-green groups together five authors, three Spanish and two Italian scholars. Ortiz Pilar has the highest link strength of seven, followed by Ortiz Rocio and Javier Becerra with six each. All these three authors are affiliated with the Spanish university Pablo de Olavide in Seville, while Marialuisa Mongelli, from the National Agency for the Development of ICT (Italy), and Roberta Fantoni, from the Italian National Agency for New Technologies, Energy & Sustainable Economic Development have rather modest collaborative ties.

The fifth cluster in violet comprises another five scholars, four of whom are affiliated with the Italian Centre for Advanced Studies, Research and Development in Sardinia: Fabio Bettio, Enrico Gobbetti, Fabio Marton, Pintus Ruggero. The fifth scholar, Holly Rushmeier, works at Yale University, and co-authored an article with Ruggero on the preservation of fragile handwriting manuscripts. The most connected author from this cluster is Gobbetti, with a link strength of 8.

Greek scholars Angeliki Antoniou (University of Peloponnese, Greece), Yannis Ioannidis (University of Cyprus), and Akrivi Katifori (National & Kapodistrian University of Athens) make another (light blue) cluster producing research on the importance of innovative pedagogies that build on digital tools to stimulate narratives and storytelling in order to spur interaction and co-learning among the visitors of cultural heritage sites (e.g., Antoniou et al., 2022).

The remaining seven clusters present only two to three interconnected authors, usually from the same institution. One of these clusters consists of Polish scholars Marek Milosz and Jerzy Montusiewicz, both from Lublin University of Technology, who co-authored three articles in our dataset, two in *ACM Journal on Computing and Cultural Heritage* and the third in *Heritage Science*.

A small but statistically significant association of 0.19 (*p* < 0.1) was found between the 78 prolific authors’ total link strength and the number of citations for their publications, which can suggest that co-authorship can increase the chance of being cited. The most cited authors on cultural heritage come from Italy, Sweden, and England, but also Australia and the US (Table 8).

**Table 8:** Top 10 most influential scholars on cultural heritage (ranked by citations).

Ranking	Author	Documents	Citations	Total link strength	Country
1	Pierdicca, Roberto	6	215	13	Italy
2	Frontoni, Emanuele	4	204	10	Italy
3	Holtorf, Cornelius	5	105	0	Sweden
4	Champion, Erik	4	67	0	Australia
5	Owens, Trevor	3	51	0	USA
6	Terras, Melissa	7	49	3	Scotland
7	Rodwell, Dennis	3	49	0	England
8	Rushmeier, Holly	3	40	1	USA
9	Lähdesmäki, Tuuli	3	40	0	Finland
10	Niccolucci, Franco	3	38	2	Italy

Authors selected based on VOSviewer output of co-authorship network analysis of the subsample of 78 authors that have at least 3 publications in the dataset.

### Co-authorship on cultural heritage at the institution level

In the next step of co-authorship networks analysis, the analyzed unit was the institution. Out of the 1747 institutions identified by VOSviewer in the dataset, 36 institutions reached the minimum threshold of eight published papers during the last 20 years. We analyzed these institutions. One needs to be cautious about the results of the co-authorship analysis at the institutional level, given the imperfect match between institutions identified in WOS and those delineated by VOSviewer with respect to the number of articles. This inconsistency derives from the numerous variants in how the author institution is referred to, not only in spelling but in whether the whole institution or different smaller research units from within said institution are referred to. Starting from the initial 1802 institutions exported from VOSviever, we reduced the discrepancies between the two files through a tedious manual search, enabling us to compare both lists of institutional names and merge the institutions with different spellings. Even if we did not eliminate all the inconsistencies from the dataset, this procedure provided a more trustworthy list, which we then used to generate a thesaurus file and imported into VOSviewer software for co-authorship analysis at an institution level.

**Fig. 3:**
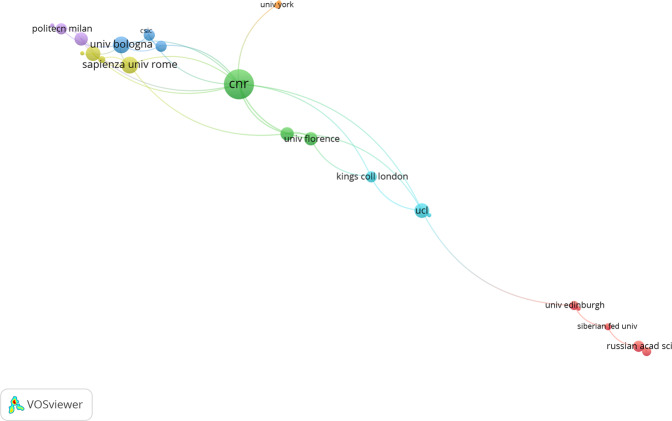
Co-authorship networks at the institution level. Note: Minimum number of papers per institution *n* = 8, number of institutions meeting this threshold *n* = 36, most connected 25 institutions are represented in the map grouping them in 7 clusters. Nod’s size indicates link strength.

Notwithstanding this shortcoming, our results show meaningful connections between 25 institutions that have co-authored at least one paper with at least one different institution. The total link strength for these institutions ranges from 1 to 11. The National Research Council (CNR), Italy, emerges as the most connected institution, co-authoring papers with other institutions, not only from Italy (e.g., University of Pisa and University of Turin), but also from elsewhere, including Spanish universities (e.g., Complutense University of Madrid), Portuguese universities (e.g., NOVA University Lisbon), and British universities (e.g., King’s College London, KCL). Figure 3 shows the seven interconnected clusters of different colors made up by two to six institutions, which vary in size according to the strength of their links.

The largest cluster in red has six items and is dominated by Russian research institutions (National Research Tomsk State University, Russian Academy of Science and Siberian Federal University). The next cluster in green consists of CNR, University of Florence, NOVA University Lisbon, and Uppsala University. The third cluster in blue is made up of two Italian universities (of Bologna and Turin) and two Spanish research institutions (The Spanish National Research Council CSIC and Complutense University of Madrid); the University of Bologna has the most connections with other universities. The fourth cluster in yellow includes the University of Curtin (Australia) and three universities from Italy, namely University of Macerata, Marche Polytechnic University and, with the most connections, Sapienza University Rome. The fifth cluster in violet comprises the Polytechnic University of Milan, University of Amsterdam, and University of Naples Federico II, the latter being the most connected. The sixth cluster in light blue groups together the KCL, University of Ljubljana and UCL. Finally, University of the Aegean, Greece, and York University, England, make up the last cluster, in orange.

For co-authorship at the institution level, there is a moderate but statistically significant correlation of 0.45 (*p* < 0.01) between the number of citations and the total link strength of an institution, as well as a rather strong statistical association of 0.72 (*p* < 0.01) between an institution’s total link strength and its number of articles in the dataset. These findings suggest that co-authorship at the institutional level brings higher numbers of citations. At the same time, the larger the number of publications on cultural heritage, the higher the chance of inter-institutional collaboration on those papers.

### Collaboration between countries

In our third analysis of collaboration, that between countries, we included countries with at least 10 publications on cultural heritage. Out of the 104 countries, 40 met this minimum threshold. The total link strength provided by VOSviewer showed that Italy has the highest number of publications on cultural heritage (291) with a total of 97 occurrences of collaborative ties with other countries. Close behind, England has a link strength of 95 identified among the 171 articles in the dataset. The least connected countries in this subsample (by link score), are Slovakia (3), Turkey (3), Argentina (2), Ukraine (1), and Lithuania (0). Germany, Switzerland, Austria, the Netherlands, and Ireland have a ratio of number of articles to link strength less than or equal to one, which could indicate that there are intense exchanges between these authors and their colleagues from other countries. These countries are more involved in cross-national cooperation in knowledge production, even if they have rather moderate number of articles on this topic, ranging from 13 articles in Ireland to 64 in Germany. By contrast, some of the very prolific countries, such as Russia, which has 126 documents on cultural heritage, displays a very poor total link strength of 3, which explains its rather isolated scientific production in this field. Eight clusters emerge from the analysis of co-authorship at a country level, indicating different patterns of cross-country collaboration. The largest cluster, in red, includes nine items (i.e., Australia, Denmark, Iran, Japan, Mexico, China, South Africa, South Korea, and ultimately Switzerland, which has the highest number of collaborative ties). The next cluster in green consists of seven countries (Croatia, England, Russia, Scotland, Slovenia, Turkey, and the US). The cluster in blue also contains seven (Germany, the most connected, with Austria, France, Greece, the Netherlands, Sweden, and Wales). The cluster in yellow consists of five countries (Brazil, Colombia, Ireland, Portugal, and Spain). Four countries make up the violet cluster (Argentina, Italy, Norway, and Romania), with Italy having the highest number of links with other countries in the co-authorship of papers on cultural heritage.

**Fig. 4:**
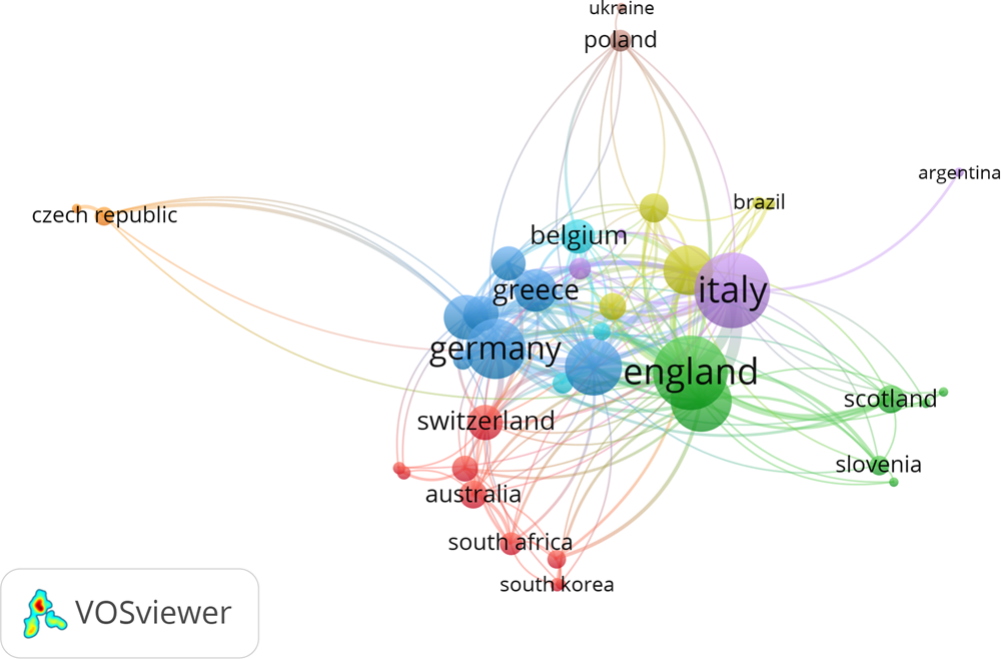
Co-authorship networks at country level. Note: Minimum number of papers per country *n* = 10, number of countries meeting this threshold *n* = 40, most connected 39 institutions are represented in the map grouping them in eight clusters. Nod’s size indicates the countries’ total link strength.

The sixth cluster, represented in light blue, encompasses only three countries (Belgium, Canada, and Finland) with Belgium being the leader as regards its total link strength. The last two clusters are similar in composition, each being constituted by two neighboring countries, namely the Czech Republic and Slovakia on the one hand, and Poland and Ukraine on the other. Although these countries share many features of their socio-historical and political past, there is hierarchy within each cluster: the second country is exclusively connected to the first, which is linked to countries in other clusters. This pattern of co-authorship is present also in the connection between Italy and Argentina in the violet cluster on the map in Fig. 4.

### Themes connected to cultural heritage and knowledge-oriented research

**Fig. 5:**
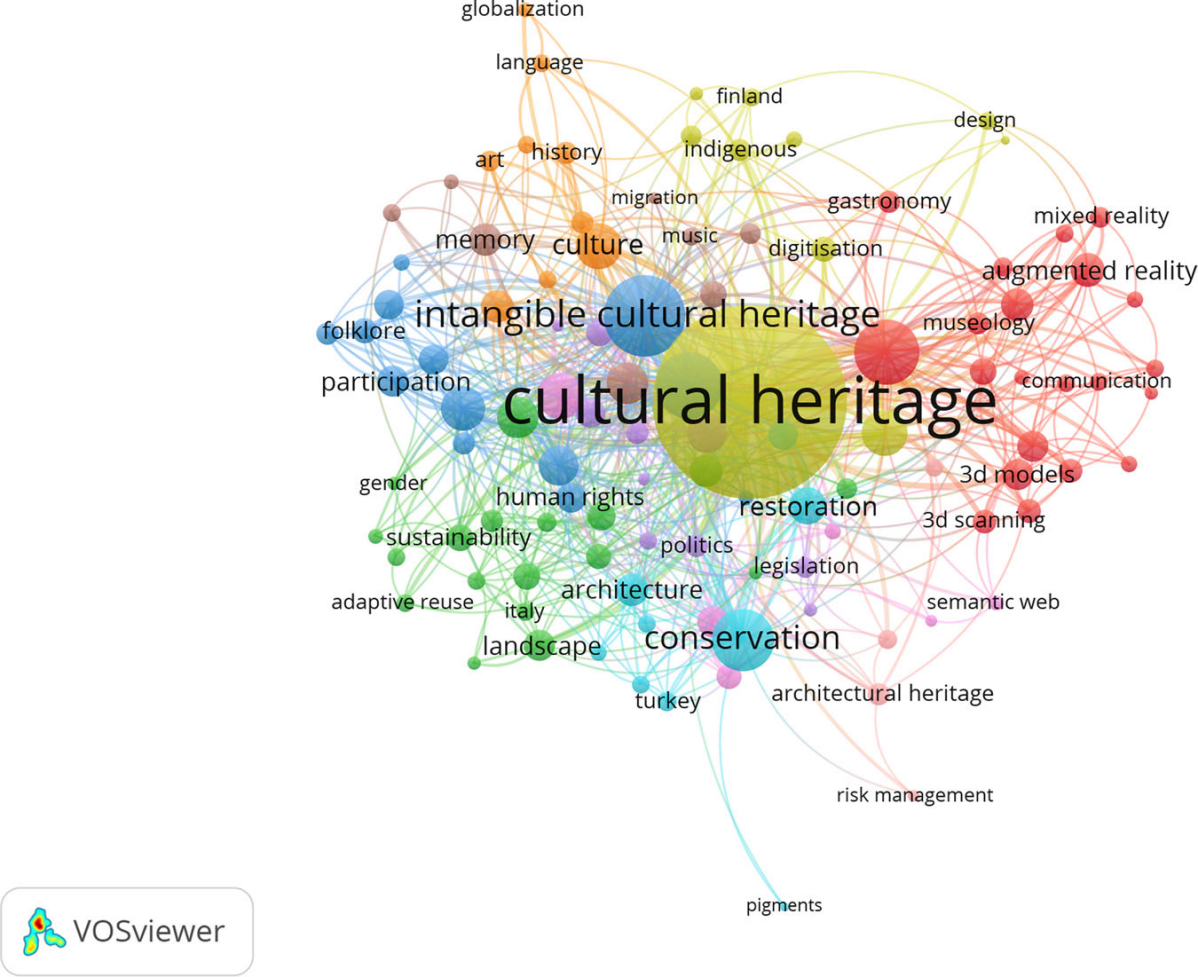
Clusters of related keywords meeting the criteria of minimum six co-occurrences. Note: each node represents a keyword sized according to its number of occurrences. Minimum number of keywords’ occurrence is 6. Nodes are connected through links that mark the co-occurrence of their attendant keywords, while the thickness of links signals the frequency of co-occurrences between keywords (i.e., the more frequently they appear together in articles, the thicker the link between two keywords).

In order to examine the main topics that authors dealing with cultural heritage write about, we performed a co-occurrence analysis of authors’ keywords in VOSviewer. We created a thesaurus file in order to merge similar words such as ‘3d model’ and ‘3d models’, ‘communities’ and ‘community’, ‘museum’ and ‘museums’, ‘digitization’ and ‘digitization’, ‘performance art’ and ‘performing arts’, and so on. Out of 6240 keywords from the full dataset, 108 words co-occurred at least six times in the list of count analysis. The normalization method of association strength was applied to the network of co-occurring author keywords. The most recurrent keywords in our dataset, co-occurring six times or more, are grouped into 10 clusters that are visually represented in different colors in Fig. 5. The dot size represents the number of times each word occurs, and the link shows the number of co-occurrences. The shorter the distance between two inter-related keywords, the more frequently they co-occur in the same articles. The most inter-related keywords in our dataset are ‘cultural heritage’ and ‘intangible cultural heritage’, with 476 and 114 link strength scores, respectively. A significant bulk of the research deals with digital aspects of cultural heritage, as shown by the largest cluster in red, which encompasses 21 keywords such as ‘virtual reality’, ‘visualization’, ‘augmented reality’, ‘3d models’, ‘3d scanning’, ‘storytelling’ and ‘serious games’, mostly in relation to museums, as this is the most inter-related keyword in this cluster. The second cluster in green brings together 18 keywords including ‘authenticity’, ‘identity’, ‘gender’, ‘resilience’, ‘empowerment,’ and ‘sustainability’; in national contexts such as Italy, China, and Japan; focusing on ‘industrial heritage’ and ‘landscape’ in connection with ‘urban heritage’. The third cluster, in dark blue, includes 13 inter-related keywords indicating a major interest in ‘heritage protection’, ‘intangible cultural heritage’, ‘community’, ‘participation’ ‘ethnography’, ‘folklore’, ‘tradition’, ‘world heritage,’ and ‘UNESCO’. The fourth cluster, in yellow, includes preoccupations with ‘digitization’, ‘social media’, ‘archives’, ‘archeology’, ‘design,’ and ‘heritage education’ among the most connected keywords alongside ‘cultural heritage’.

Structural and institutional features regulating the cultural heritage domain are dealt within research using specific keywords, such as ‘national identity’, ‘legislation’, ‘politics,’ and ‘cultural politics’, often discussed in relation to ‘European Union’ and especially European countries that are typical destinations of ‘cultural tourism’, such as Spain and Greece, indicating a growing concern for ‘cultural heritage management’. These keywords are united in a fifth cluster, in violet, with 10 keywords. The sixth cluster in light blue connects nine keywords that reveal researchers’ growing contribution to material or tangible cultural heritage in the form of ‘built heritage’, ‘historic buildings’ requiring ‘restoration’, conservation’ alongside the intangible ‘values’ and ‘education’, with some emphasis on Turkey as a context requiring a special attention to values, human rights, and management of its archeological sites.

The seventh cluster of keywords, presented in orange, includes rather soft items such as ‘art’, ‘culture’, ‘language’, ‘history’, ‘living heritage’ that may be affected by ‘globalization’. In the eighth cluster, in brown, with nine keywords, ‘monuments’ and Poland provide some materiality and context to the scientific production on cultural heritage; ‘cultural memory’, ‘migration’, ‘digital cultural heritage’, ‘music’, and ‘cultural landscape’ show how the preservation of memory is an integral part of cultural heritage; other themes include the impact of the ‘COVID-19 pandemic’ on ‘cultural tourism’ and its management, as well as the effects of ‘climate change’ on ‘built heritage’. Finally, the tenth cluster in light brown groups four inter-related items on ‘architectural heritage’ and the policies of ‘risk management’ to ensure ‘protection’ and ‘reconstruction’ of cultural heritage.

**Fig. 6:**
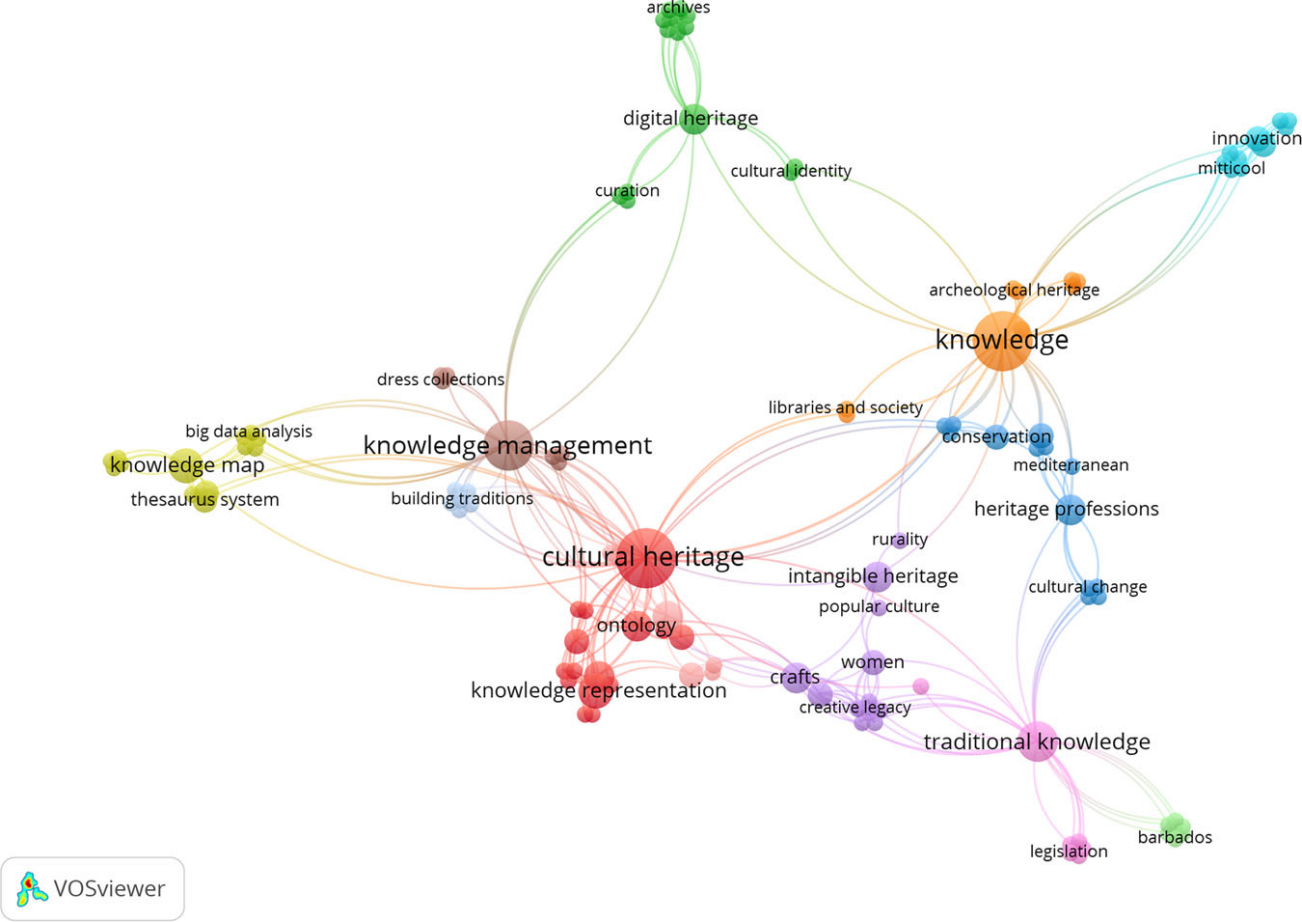
Clusters of co-occurring keywords related to knowledge. Note: VOSviewer co-occurrence analysis of 119 author keywords present in the articles containing terms referring to knowledge. Node size reflects the number of occurrences of keywords. Nodes are connected through links that mark the co-occurrence of their attendant keywords, while the thickness of links signals the frequency of co-occurrences between keywords (i.e., the more frequently they appear together in articles, the thicker the link between two keywords).

During the past decade, some scholars (e.g., Smith, 2006) have pinpointed how cultural heritage is about knowledge production of the past, present, and future, and also about who ‘we’ and ‘others’ are. In our analysis of the subsample of 37 articles that include some reference to knowledge in the author keywords, we identified 119 keywords related to this relatively new cultural heritage research subfield. These are organized in 12 clusters (Fig. 6) revolving around prominent terms such as ‘knowledge management’, ‘knowledge representation’, ‘knowledge map’, and ‘knowledge mobilization’. The first largest cluster include 15 keywords such as ‘knowledge representation’, ‘semantic web’, ‘ontology’, ‘diversity’, ‘open data’, ‘fine art’, and ‘legacy data conversion’. The second cluster consists of 14 co-occurring keywords, among which the most prominent in terms of their total link strength are ‘digital heritage’, with a total link strength of 15, followed by ‘archives’, ‘copyright’, ‘heritage’, indigenous cultural material’, ‘knowledge mobilization’, ‘provenance’, ‘repatriation’, and ‘repositories, with a total link strength of 8, indicating moderate levels of co-occurrence with other keywords. The third keyword group includes themes related to ‘heritage professions’ and ‘conservation’, while paying attention to aspects linked to ‘education’, ‘values’ and ‘cultural change’, with an emphasis on knowledge about ‘minority groups’ and ‘ethnic identity’. The next cluster of interconnected keywords from knowledge-oriented articles deals with ‘crafts’, ‘women’ and their’empowerment’, ‘creative legacy’, and ‘cultural expression’, as well as with aspects regarding ‘social inequality’, ‘popular culture’, and ‘rurality’. The relationship between cultural heritage and knowledge is also studied through ‘innovation’, ‘sustainable development’, ‘underwater cultural heritage’. Another way to engage with knowledge among researchers focused on cultural heritage is to connect knowledge to ‘information and development’, ‘libraries and society’, and ‘memories’, as suggested by prominent keywords from the seventh cluster. Another strand of research deals with ‘knowledge management’ and ‘heritage impact assessment’, alongside ‘ethnography’, ‘dress collections’, and ‘fashion’. Likewise, some researchers explore the links between ‘traditional knowledge’, ‘indigenous data sovereignty’, and ‘legislation’. Knowledge-related research on cultural heritage also considers the aspects of ‘semantics’, ‘narratives’, ‘storytelling’, alongside ‘image retrieval’ and ‘augmented reality’. Finally, the last two keyword clusters stemming from the knowledge-specific research on cultural heritage focus on the connections between the performance of ‘identity’ and ‘funerary traditions’ set in ‘Caribbean culture’, and on ‘citizen science’, ‘vernacular architecture’, and the development of ‘web and mobile applications’ for the study and consumption of cultural heritage, respectively.

Our analysis reveals that only a handful of scholars have started to include these knowledge-related terms in their scientific production, which means that the level of awareness about the assimilation of the knowledge-oriented approach in the study of cultural heritage is still in its infancy.

The earliest article in this subsample dates back to 2009 and examines the moral knowledge in Turkey in relation to value teaching and religious culture (Taşdemir, 2009). Articles dealing with the relationship between cultural heritage and knowledge have gradually increased in number since then, reaching a peak of nine articles in 2021, written mostly by authors located in Italy, Germany, and Greece (where nine, six, and five articles contain phrases related to knowledge, respectively).

Knowledge-oriented research has a high potential for innovation in the field of cultural heritage, since it brings new underexplored themes to this field, such as the contribution of women and various vulnerable minorities to existing knowledge on cultural heritage, as well as their participation in its preservation. Knowledge-oriented research also highlights the heritage professions and the potential of big data to create knowledge maps of cultural heritage. All these represent promising avenues for researchers.

## Discussion in the context of recent developments in cultural heritage

The results of our analysis reflect the rapid change in the cultural heritage field since the 2000s. These changes include the technological development of systems and tools used for conserving, preserving, and managing cultural heritage, as well as the digitization of various basic functions of museums, archives, and libraries including the identification, organization, storage, and dissemination of information. Moreover, institutions dealing with cultural heritage have boldly tested and put into service virtual and augmented reality applications and enhanced their exhibitions and audience work through gamification. The increasing awareness of the climate crisis and the need for sustainability measures in the last two decades has broadly impacted the cultural heritage field. These concerns are closely connected to social inequality and exclusion. Heritage institutions have sought to respond to these concerns through community-oriented projects, encouraging bottom-up initiatives and facilitating the participation of diverse population groups.

As indicated by our analysis of the themes connected to cultural heritage and knowledge-oriented research, WOS-indexed cultural heritage research actively tackles the above-mentioned changes and timely challenges and concerns that impact not only the cultural heritage field but society more broadly (Su et al., 2019; Schmid, 2020). Moreover, the results of the co-occurrence analysis of authors’ keywords reflect the development of international cultural heritage governance and management and the focus points of international heritage conventions and charters from the past two decades, ranging from digital to intangible cultural heritage and from landscapes to the economic and social value of cultural heritage for society. The results underline the societal relevance and timeliness of cultural heritage research, particularly during the past five years containing over 80% of WOS-indexed articles in our data.

The analysis of publication volume and production patterns in terms of co-authorship, collaboration, citation, and keywords reflects the current paradigmatic emphasis and power relations in cultural heritage research. In the 2000s, cultural heritage embraced a new critical research paradigm. Scholars have become increasingly interested in complex questions regarding the power entailed and produced by heritage among and between people, communities, and societies (Ashworth et al., 2007; Waterton and Smith, 2009; Mydland and Grahn, 2012; Logan, 2012; Harrison, 2013a; Lähdesmäki et al., 2019). The interdisciplinary field of critical heritage studies has emerged in order to address uneven power relations, hierarchical power structures, explicit and implicit politics of dominance and oppression, silenced narratives, and alternative, emancipatory, and empowering identity projects based or drawing on cultural heritage (Lähdesmäki et al., 2019). Through research interests of this kind, the conception of cultural heritage has been extended to include political, societal, and ideological meanings, as well as dissonant and contested dimensions (Kisić, 2017; van Huis et al., 2019). Our results on the co-occurrence of keywords and the host countries and institutions of the most actively publishing scholars reflect this paradigm change in cultural heritage research. Critical heritage studies have been strongly developed by scholars from English and Australian universities—including Harrison and Winter. As shown in Table 7, most cited articles include critical stances regarding the suitability of mainstream ‘western’ approaches to heritage preservation on different continents (Winter, 2014), or the lack of thoughtful consideration by heritage practitioners who, when they conduct their work as a purely technical endeavor, decoupled from the political and social contexts in which communities live (Chirikure et al., 2010; Logan, 2012), obliterate human rights and communities’ identities. Other influential works on cultural heritage focus on micro-processes of ascribing value to popular music, as opposed to authorized discourses on music heritage in the UK (Roberts and Cohen, 2014), or on the voluntary work conducted by laypeople with no professional background dedicated to the maintenance of traditions, which is not listed as heritage by authorities in Norway but is seen as critical for local communities’ identity (Mydland and Grahn, 2012). Power asymmetries in establishing the worthiness of being officially acknowledged and celebrated as heritage are therefore hotly debated and attract scholars’ interest, as reflected in the number of citations. Other highly cited articles study the damaging impact of climate change on built heritage (Leissner et al., 2015) or the use and effectiveness of digital games in facilitating the acquisition of historical knowledge by teenagers in Italy involved in experimental research design (Rubino et al., 2015).

Despite the critical scholars’ aim to break with a Eurocentric tradition and with a Western focus in cultural heritage scholarship (Waterton and Smith, 2009; Winter, 2014), our results show that WOS-indexed cultural heritage research is still very much biased towards scholars from European countries and research institutes. Moreover, our analysis shows how European scholars and institutes actively collaborate among other. Such results can be partially explained by the funding of their research projects. In our data, the European Union, with its various funding programs, was the most acknowledged financer or co-financer of research (in 117 articles), followed by UK Research and Innovation UKRI (43), and the Arts and Humanities Research Council (33). During the 2000s, the European Union has increased its emphasis on cultural heritage as a policy tool that is expected to have a broad positive impact on European societies (Lähdesmäki et al., 2020). This emphasis is reflected in European research funding seeking to strengthen heritage conservation and protection and heritage-related innovations in Europe, as well as intercultural dialog and participation in European societies. The European Union invested around €100 million in heritage research between 2007 and 2013 through its Seventh Framework Program (Zabeo and Pellizzon, 2017), and increased this funding to €500 million between 2014 and 2021 in the next program, Horizon 2020 (EC European Commission, 2022). The emphasis on cultural heritage research continues in the current funding program. The key criteria for the European Union’s research funding are multidisciplinary collaboration between European scholars and research institutes producing high-quality research, disseminated effectively through open access publications. Other major European research funders, such as UKRI, value similar features (multidisciplinary international collaboration, excellence of research, and open access). Such funding criteria have a major impact on cultural heritage research in general and strengthen its Eurocentric profile.

## Conclusions

Our article contributes to cultural heritage research by providing critical knowledge on structural aspects shaping the publication volume and production patterns in multidisciplinary humanities exploring cultural heritage. The scholarship had previously lacked such knowledge, although publication volume and citation metrics are currently important parameters in assessing researchers’ performance in humanities as well. Besides such knowledge, the article contributes to broadening methodology in the scholarship of cultural heritage, which includes only a few earlier studies drawing on bibliometrics. At the same time, the methods and techniques of bibliometric analysis underline the article’s theoretical contribution to the scholarship: the analysis brings forth various interdependencies drawing on authorship, co-authorship, research collaboration, institutional affiliation, countries of affiliation, citation, and research funding. We summarize our key results in the following.

Our study shows how humanities research on cultural heritage is a broad and multidisciplinary field covering topics that reflect technological, social, and environmental changes, the adoption of international heritage conventions, and the deepening of knowledge in the scholarship during the past 20 years. Recently, publication productivity of WOS-indexed journal articles on cultural heritage has steeply increased, while the few leading journals in the field have strengthened their position as preferred and sought-after dissemination fora for research results. In our study, the leading journal in terms of publication quantity was the *International Journal of Cultural Heritag*, which has an interdisciplinary profile and welcomes critical contributions and debates on the nature and meaning of heritage. In such critical contributions, cultural heritage is often considered as a complex process of knowledge production. Our study indicates, however, that such an approach to cultural heritage is still underdeveloped in WOS-indexed journal articles.

In cultural heritage research, practices have become more collaborative, not least due to funding criteria. Our analysis shows, however, that cultural heritage researchers often collaborate in small teams affiliated with the same institution. Based on our analysis, international collaboration and co-authorship increase the chance of being cited and, implicitly, the scientific impact. This result aligns with previous research noting how publication volume is strongly associated with international collaboration (Abramo et al., 2009; Kyvik and Aksnes, 2015; Fursov et al., 2016) and receiving research funding (Kyvik and Aksnes, 2015; Wahid et al., 2022).

Our study points towards the Eurocentrism of cultural heritage research indexed in WOS. The results show how the authorship of WOS-indexed journal articles concentrates on a limited number of institutions and countries: Italian, English, and Spanish scholars are the most productive authors. Likewise, the most cited scholars in our data were Europeans (from Italy and Sweden). These figures on quantity do not paint the full picture of high-quality cultural heritage scholarship. Our study underlines the nature of WOS as a tool of knowledge management in academia: it organizes information on cultural heritage research by structuring it into categories and research areas which such multi- and interdisciplinary research is difficult to fit into. As one of the core global providers of publication volume and citation data, WOS has an impact on the image of esteemed cultural heritage research, as well as on scholars’ understanding of their own field. WOS itself can be seen as a Western platform continuing the Eurocentric history of science (Poskett, 2022).

Our study naturally has its limitations. We have focused our analysis on peer-reviewed journal articles, albeit many humanities scholars still consider monographs and edited volumes as the most respectable way of publishing research results. Furthermore, we limited our analysis to articles written in English or including a title, keywords, and/or abstract in English. Even though English is the contemporary *lingua franca* in academia, many non-English-speaking scholars in cultural heritage research want to publish their results in their mother tongue, particularly when researching local, regional, or national case studies in order to serve the researched communities.

Our results and the identified limitations of the study underline various challenges in cultural heritage research and WOS. The key challenge faced by research is broadening the field in order to include various voices and views from all continents in its knowledge production. Cultural heritage research would benefit from a more active cross-continental collaboration of scholars and research institutes. In general, Eurocentrism and the focus on Western academia are central traits displayed by WOS. Its key challenge is to more generously acknowledge high-quality publications conducted globally in various humanities fields and in various languages.

Based on our bibliometric analysis, we have formulated suggestions for developing the scholarship in cultural heritage research. First, international collaboration and co-authorship are likely to increase citations and therefore pay off in terms of research impact. Second, strengthened collaboration between scholars from other continents will deconstruct the Eurocentrism of cultural heritage research, diversify research topics, and increase the multitude of voices in research outcomes. Third, we suggest applying bibliometric methods and approaches in the analysis of more specific cultural heritage topics in order to illustrate how such research is produced, by whom, and where. Finally, we suggest exploring the complexity of knowledge production in cultural heritage in order to open new research avenues.

## Data Availability

Data sharing is not applicable to this article as no datasets were generated during the current study that is based on bibliometric information on published articles from Web of Science.
